# Migration, Embeddedness, and Vulnerability During the COVID-19 Pandemic

**DOI:** 10.1007/s12134-023-01015-x

**Published:** 2023-03-27

**Authors:** Kevin Patrick O’Dell, Sonja Fransen, Dominique Jolivet

**Affiliations:** UNU-MERIT, Maastricht University, Maastricht, the Netherlands

**Keywords:** COVID-19, Migration, Local embeddedness, Vulnerability, Inequality, Amsterdam

## Abstract

The COVID-19 pandemic and concomitant policy measures have disproportionally affected the lives of migrants worldwide. Focusing on inequalities between social groups, studies have tended to neglect the role of local embeddedness as a factor influencing the extent to which individuals are affected by COVID-19. In this paper, we study the vulnerabilities of people with different migration experiences in an urban setting in the early stages of the pandemic, focusing on three key livelihood assets: economic, social, and human capital (health). Our analyses are based on online survey data (*n* = 1381) collected among international migrants, second-generation residents (those with at least one parent born abroad), and non-migrants residing in Amsterdam in July 2020. We find that international migrants, and particularly those who arrived in the city more recently, reported larger shocks to their economic and social capital than other city residents. This finding illustrates the vulnerabilities of “newcomers” to the city and their limited resilience to shocks. Second-generation residents were particularly vulnerable in terms of health, but this relationship was strongly mediated by education and neighborhood effects. In all three groups, those with poor relative wealth and those who were self-employed were more vulnerable to economic shocks. Our findings illustrate how the COVID-19 pandemic has exacerbated inequalities in vulnerabilities across migrant and non-migrant groups, and how those who were locally embedded, including migrants and non-migrants, were less likely to be negatively affected by the pandemic.

## Introduction

The COVID-19 pandemic and the policy measures that have been implemented to curb the spread of the virus have affected the lives of individuals globally. However, migrant and refugee populations may be particularly vulnerable to the direct and indirect impacts of the pandemic because of their—varying degrees of—economic, social, and political exclusion in host countries (Guadagno, 2020). An increasing body of literature is emerging on the impacts of the pandemic on migrant populations and refugees (Antonides & van Leeuwen 2020; Carpentieri et al., 2020; Ćudić et al., 2020; Germain & Yong, 2020; Gottlieb et al., 2020; Machado & Goldenberg, 2021; Visser et al., 2020). Overall, this literature has shown that migrants often work in more precarious jobs, have fewer social networks, and may even lack proper access to basic services such as healthcare. As such, migrants may be more prone to income insecurity, psychosocial problems, and health problems during the pandemic. Migrants are also likely to face language and cultural barriers (Guadagno, 2020; Kluge et al., 2020), which limits their access to information and support. Given these additional vulnerabilities, the pandemic is likely to exacerbate existing inequalities between migrant and non-migrant populations in various settings.

Despite these important observations, existing studies on COVID-19 and migrant experiences have considered migrants (and non-migrants) as rather homogeneous groups without taking into account important factors such as the time spent in the place of residence or the strength of their social networks when coping with the adverse effects of the pandemic. Moreover, studies distinguishing between migrants and non-migrants tend to assume the lack of migration experiences of so-called non-migrants. Non-migrants could be born in the country of residence but could have migrated. Non-migrants could also have some migration experience when they emigrated and then returned to their place of birth. As migrants, non-migrants could also have translocal lifestyles, which could affect their length and degree of social and economic interaction in their main place of residence.

To consider this diversity in migration experiences, we study how the local embeddedness of city residents—migrants and non-migrants—affects their vulnerabilities during the COVID-19 pandemic. Embeddedness is a concept that has its origins in the economics literature and that was originally used to study how social structures impact economic actions and performance (Granovetter, 1985; Polanyi et al., 1957). In the migration literature, embeddedness has been applied in studies on immigration and integration (Portes & Sensenbrenner, 1993; Ryan & Mulholland, 2015; Wessendorf & Phillimore, 2019), migrant entrepreneurship (Barberis & Solano, 2018; Kloosterman, 2010; Kloosterman et al., 1999; Solano, 2020), and studies on return migration (Ruben et al., 2009). Here, we build on the individual conceptualization of embeddedness developed by Ruben et al., (2009, p. 910) that refers to “the ways how individuals find and define their position in society, feel a sense of belonging and possibilities for participation in society,” and that includes three interrelated dimensions: economic, social network, and psychosocial embeddedness. We study how these three dimensions of embeddedness affect the extent to which individuals with diverse migration experiences are negatively impacted by the COVID-19 pandemic, hypothesizing that the level of their embeddedness decreases vulnerabilities during the pandemic. To measure vulnerability, we apply an asset vulnerability framework designed for urban areas (Moser, 1998) and focus on three key assets that could have been affected by the pandemic: economic, social, and human capital (health).

We focus our study on international migrants, second-generation residents (those born in the Netherlands with at least one parent born abroad), and non-migrants (those born in the Netherlands with both parents born in the Netherlands)—in an urban, high-income environment: the city of Amsterdam. Amsterdam makes for an interesting case study because it hosted more than 180 nationalities and consisted for 55.6% of individuals with a migration background in 2020 (CBS 2020). The city’s economy was hit hard by the pandemic, because of the relatively large presence of sectors such as catering, aviation, accommodation, plus wholesale, and retail trade. Figures published by Statistics Netherlands in August 2020 showed that the Amsterdam economy had shrunk by 12 to 14% since the beginning of the pandemic, as compared to a national decline of 9.3%. The economic downturn quickly translated into rising unemployment: the number of individuals receiving unemployment benefits in Amsterdam increased by 61% in July 2020 compared to July 2019 (OIS 2021b). Additionally, the number of sold houses in Amsterdam decreased by 7.4% in June 2020 compared to June 2019 (OIS 2021b). Most migrants work in transport, industry, and real estate sectors—which made them relatively more vulnerable to the economic risks related to COVID-19 (Gemeente Amsterdam, 2021b).

Our survey was data collected in July 2020, approximately 4 months after the start of the first “lockdown” in the Netherlands. Data was collected online, in collaboration with Research, Information, and Statistics (Onderzoek, Informatie en Statistiek—OIS), the Amsterdam municipality department that regularly conducts online research through its city panel.^1^ A total of 1381 individuals participated in the online survey and answered questions about their lives before and after the pandemic. Our three groups of survey respondents are comparable in terms of education levels (mainly people with bachelor or master level) and proficiency in Dutch,^2^ which allows us to study how economic, social, and psychosocial embeddedness may play a role in reducing vulnerabilities against the negative impacts of the pandemic across groups with diverse migration experiences.

Our findings show how economic, social, and psychosocial embeddedness play an important role in the extent to which respondents are affected by the pandemic. Particularly in terms of economic vulnerability, we find that those with higher levels of embeddedness were less likely to have lost income due to the pandemic. Psychosocial embeddedness, on the other hand, plays an important role in reducing the social and health-related impacts of the pandemic. In line with previous studies, we find that the pandemic does exacerbate existing inequalities between groups with different migration backgrounds, but our results highlight how these impacts are moderated by other socio-demographic variables such as education level, occupational status, or relative wealth. Moreover, we find that the different vulnerabilities (economic, social, and health-related) that individuals experience during the pandemic are highly related and mutually reinforce each other.

## COVID-19 and (Migrant) Vulnerability

Empirical studies on the impacts of COVID-19 on migrants have examined how existing vulnerabilities—in terms of mental health, physical well-being, or employment—were exacerbated during the first outbreak. Some of the earliest studies on the pandemic’s effects were conducted in the Asia–Pacific region where the first COVID-19 cases were reported. Specifically in China, research focused on internal migrants who may have been some of the earliest transmitters during their transit from urban workplaces to rural homeplaces (Liu et al., 2020). This research runs parallel with studies that focused on institutional inequalities, and particularly the role of biases, misguided arguments, and even racialization of migrant groups among governments and healthcare practitioners, which led to worse health and well-being among migrants (Germain & Yong, 2020; Gottlieb et al., 2020; Guo et al., 2020; Machado & Goldenberg, 2021). Other studies focused on volatile employment conditions among migrant groups (e.g., Ćudić et al., 2020), showing how a lack of institutional protection available to unskilled migrant workers, who would be considered already vulnerable in economic terms, increased their economic vulnerability.

Generally, migrants are regarded as a minority group with worse health, economic, and social outcomes since the pandemic’s onset. An important aspect that increases the vulnerabilities of migrant populations relates to access, particularly, a lack of equitable access to healthcare and welfare services that leads to unfavorable outcomes for migrant groups. The lack of access to services can be due to administrative barriers, financial barriers (Germain & Yong, 2020), or psychosocial factors. The International Organization for Migration (IOM, 2020), for example, found that migrants with irregular status were reluctant to seek care because they fear deportation (Guadagno, 2020). Ngiam et al., (2021) found that migrant workers in Singapore were not necessarily barred from hospital care, but were instead confined to migrant dormitories and could not access the city-state’s broader housing market. Overall, the literature makes a case that disparities in terms of access to social services need to be rectified in the spirit of ensuring equal rights for all groups.

### The Role of Embeddedness in Vulnerability

The abovementioned studies tend to focus on the vulnerabilities that migrants face compared to non-migrants. Such vulnerabilities are explained by migrants’ insecure jobs in secondary labor markets, their administrative status, and their limited access to welfare state resources. In sum, emphasis is placed on the more tangible and material structural conditions that shape their level of vulnerability during the COVID-19 pandemic. Less is known about vulnerabilities caused by the interplay between such structural factors and meso- and micro-level factors, some of them linked to migration experiences. Moreover, less tangible aspects such as social networks are explored in less detail.

In this study, we apply the concept of embeddedness to capture how the depth of relations between an individual and his or her local surroundings may reduce vulnerabilities to shocks such as the pandemic. As such, our perspective on embeddedness is closer to Granovetter’s (1985) focus on the meso-level than to Polanyi’s (1944) macro-level lens, as we focus on the local, community-level embeddedness of individuals. We perceive the concept of embeddedness to be particularly suitable to study the local experiences of individuals with a diverse range of migration experiences. As Ryan and Mulholland (2015, p. 136) describe, “Embedding offers ways of thinking about the nuanced details of migrants’ experiences of engagement with the people and places that make up their social world, and in a way that may mitigate often fixed and narrow concepts such as ‘integration.’” Embeddedness is considered a dynamic concept, as people’s experiences in a particular place are constructed through their interaction with society over time (Findlay & Stockdale, p. 10). As such, embeddedness is a dynamic process of connection and interaction at social, economic, and political level (Ryan, p. 142).

Embeddedness is generally described as a multidimensional concept. Hess (2004), for example, defined three dimensions of embeddedness, including network or relational embeddedness, societal or cultural embeddedness, and territorial embeddedness, which, together, determine the context, in a specific time and place, in which individuals function as socio-economic actors. Taking into account this multidimensional and local context is important, as it prescribes the structural opportunities and obstacles that migrants may face (Ryan, 2018). As described, we focus on local embeddedness of individuals, composed of three dimensions, which include economic, social network, and psychosocial embeddedness (Ruben et al., 2009). Whereas economic embeddedness relates to tangible and material aspects such as employment or asset ownership, social network embeddedness refers to the social relations and information that an individual has access to. Finally, psychosocial embeddedness refers to the extent to which an individual feels integrated and safe in a certain locality.

We hypothesize that those who are more embedded in the city of Amsterdam and/or their neighborhoods are less vulnerable during the COVID-19 pandemic. Vulnerability can be understood as the insecurity and sensitivity in the well-being of individuals and households, independently of their socio-economic positions, when they are confronted to sudden shocks, long-term trends, or seasonal cycles, as well as their responsiveness and resilience to the subsequent increased risks and uncertainties (Moser, 1998, 3). This framework has been used in various settings to measure vulnerability of households and individuals during shocks and disasters (Afriyie et al., 2018; Busetta et al., 2021; Chiwaula et al., 2011; O’Loghlen & McWilliams, 2017). Although the framework has been applied mostly in poorer settings, it is nevertheless a useful tool to study the multidimensionality of vulnerability in high-income settings as well, due to its multidimensional approach and the focus on both tangible assets (e.g., income, housing) and non-tangible assets, such as human capital and social networks.

**Fig. 1:**
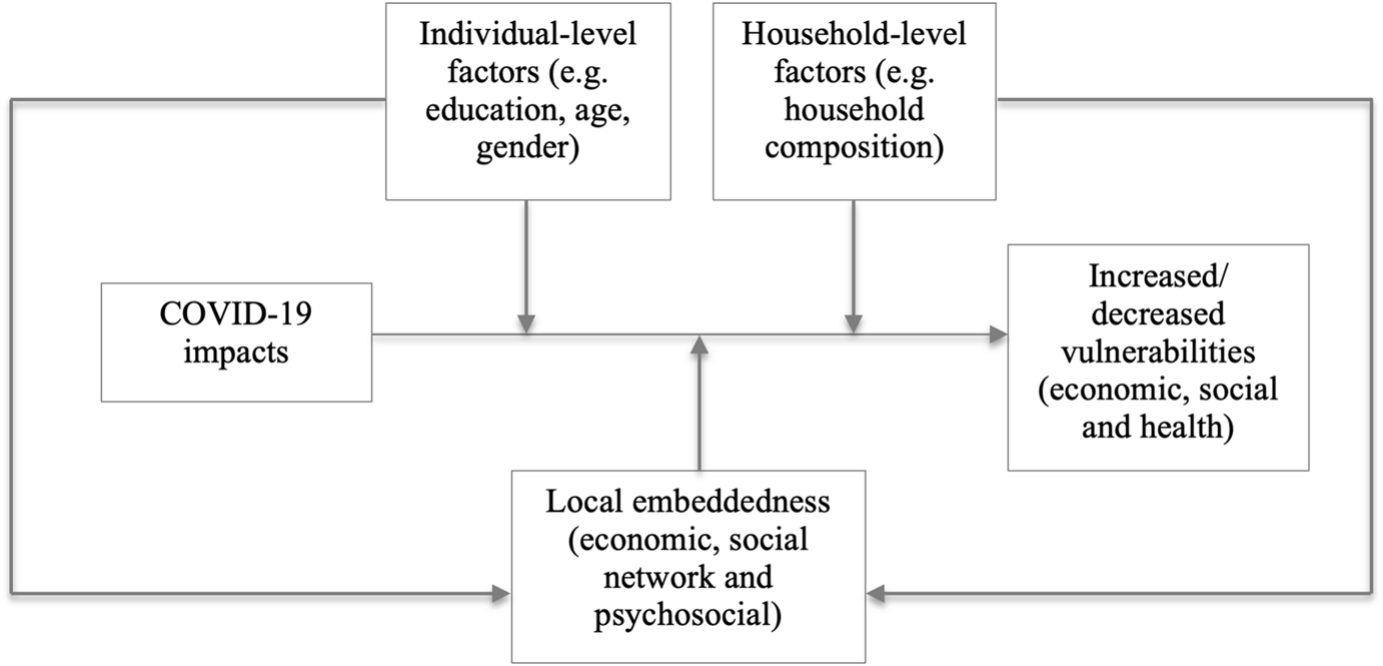
Conceptual model: effects of the COVDI-19 pandemic and local embeddedness

We also assume that, in combination with local embeddedness, vulnerabilities of individuals in a particular place can vary according to other characteristics such as gender, the size of their household, or their degree of dependency on the labor market as main source of income. Within the Dutch welfare state, a pensioner, for instance, is more likely to maintain the same level of income in times of economic recession than somebody who is self-employed. We therefore incorporate in our analysis individual and household-level factors (Fig. 1). At individual level, we take into account the effect that the level of education and the position in the labor market (employee, self-employed, unemployed, retiree) in shaping the effects of the COVID-19 pandemic. At household level, Moser (1998) stressed the key role of household relations in shaping how people respond to and cope with risks and shocks. Therefore, we incorporate into our framework household composition as a factor to evaluate the effects of the pandemic in people’s level of vulnerability. Finally, we add local embeddedness in the city of residence.

## Case Study: COVID-19 and the City of Amsterdam

**Map 1:**
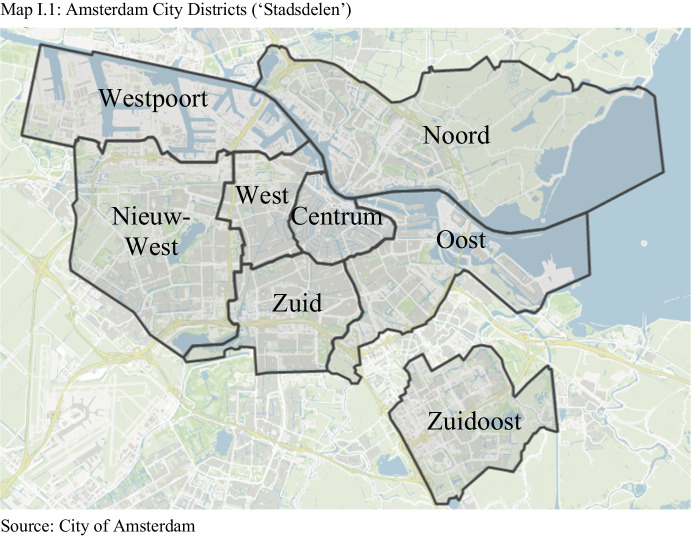
Amsterdam city districts (“Stadsdelen”). Source: City of Amsterdam

We focus our study on the city of Amsterdam, a highly diverse city, located on the Amstel River in the North (Noord) Holland province. Amsterdam has a rich migration history (Bosma, 2012; Vermeulen & Van Heelsum, 2012)—especially during the Dutch post-colonial period in the latter half of the twentieth century, when the country received immigrants with backgrounds ranging from Moluccan and Indonesian to Afro-Caribbean and Surinamese. In 2020, the city hosted more than 180 nationalities and consisted for 55.6% of individuals with a migration background as compared to a national average of 24.8% (CBS 2020). Over the past years, newly arrived migrants with study, work, or entrepreneurial visas consisted mostly of Indian, American, and Chinese nationals while family visa were primarily granted to Indian, Syrian, and Turkish Nationals. The city is divided into eight administrative districts which each contain their own subsets of neighborhoods (see Map 1 of the Appendix).

Table 1 gives an overview of the Amsterdam districts and their characteristics. Although all neighborhoods host diverse populations, the districts with the highest percentages of residents with a migration background are Southeast and New-West. These districts are not the most densely populated areas of the city, but do host higher percentages of households that are considered poor, measured in this case as annual income under €24,793 per year.

**Table 1:** Amsterdam districts (“Stadsdelen)”: population density and diversity in 2020

City district	Surface area per district (%)	Population density per km^2^	Housing density per km^2^	Population with a migration background (2021) (%)	Households in poverty, income less than €24,793 per year (2018)1 (%)
Centrum	4	13,903	8754	46	13
West	5	15,600	8504	53	17
N.-West	17	4726	2055	69	19
South	8	9469	5298	47	12
East	14	7985	4069	52	16
North	29	2377	1101	52	20
Southeast	10	4492	2100	75	24
Total/average	100	5296	2715	56	17

Source: Oppervlakte, Bevolkingsdichtheid En Woningdichtheid Naar Stadsdelen /OIS, January 2020. Bevolking naar burgerlijke staat en geslacht/OIS, 2021. Stadsdeelrapportage Amsterdamse Armoedemonitor 2019. Data is scarcely available for the industrial district Westpoort, and this district is therefore excluded from this table

^1^Entire year’s income; excluding students and institutions

### COVID-19 and Its Impacts in Amsterdam

On 17 March 2020, the Mayor of Amsterdam announced a set of measures to contain the coronavirus, including the closure of schools, restaurants, and cultural venues. While similar lockdown measures were implemented across Western European capitals, Amsterdam’s approach, which aligned with the national Dutch strategy, deviated from those of other European cities because no strict enforcement was used; residents were expected to act responsibly (Antonides & van Leeuwen 2020). In practice, the government prioritized economic recovery and elderly safety in what they called a “smart lockdown” that lacked strict stay-at-home orders seen in countries like Spain and Italy (Antonides & van Leeuwen 2020). Retrospective studies of the city’s lockdown identify the elderly as the primary vulnerable population (Carpentieri et al., 2020; Visser et al., 2020), while the city’s young residents also reported decreased well-being due to reduced social contact (Antonides & van Leeuwen 2020).

**Fig. 2:**
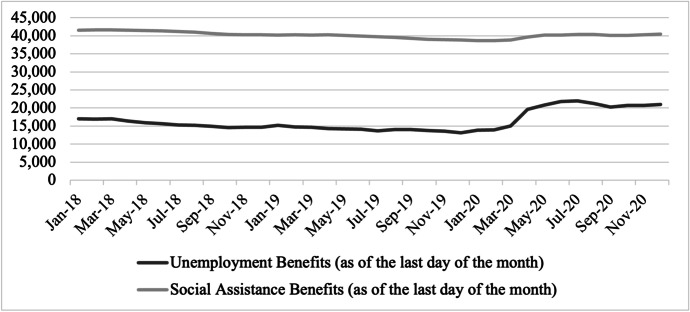
Amsterdam Residents receiving unemployment and social assistance benefits, 2018–2020. Source: Kerncijfers Amsterdamse Economie update March 2021

The impact of the lockdown is most visual in statistics on unemployment and social assistance benefits received in the city (Fig. 2). Unemployment benefits are typically applicable to individuals finding themselves temporarily unemployed whereas individuals in a long-term state of unemployment (longer than a year) typically receive social assistance benefits. We observe that the numbers of unemployment and social assistant benefit recipients increased between the end of March and June 2020, which suggests that many individuals lost their jobs during the pandemic. Studies have shown that individuals with a migration background, and particularly those with a non-Western migration background and those who were lower educated, were more likely to lose their jobs due to the pandemic and to receive unemployment benefits (Burema et al., 2020).^3^ Even in employment sectors that were hit hardest by the lockdown, such as the catering industry, retail, and financial services, people with a migration background were more affected than those without a migration background, because individuals with a migration background are more likely to have lower paid jobs and flexible contracts within these sectors. During the crisis, young people with a migration background were also more likely to lose their jobs than young people without a migration background.

Amsterdam residents with a migration background also experienced more significant health impacts of COVID-19 than other city residents (GGD Amsterdam, 2021a, b). They were more often admitted to the hospital, had higher chances of getting infected by the virus, and had higher mortality rates if affected. The researchers attributed this to the low socio-economic status of certain migrant groups, their underlying health conditions, living conditions (residing in smaller apartments with larger families), working conditions, language difficulties, and lack of suitable communication from the municipality targeted to these groups.

While these previous studies provided important insights into the impacts of corona on migrants and non-migrants in the city, most studies focused on the most vulnerable groups of migrants and emphasize how their existing vulnerabilities, e.g., in terms of socio-economic status and living conditions, made them more vulnerable to the impact of the pandemic. Our study builds on these insights by focusing on another factor that may affect people’s vulnerability during the COVID-19 pandemic—their embeddedness in Amsterdam.

## Data and Methods

Our data consists of surveys collected in the city of Amsterdam in July 2020 through an online panel survey. The survey was formulated and distributed in collaboration with OIS Amsterdam (Research, Information and Statistics), which is an organization that is part of The City of Amsterdam that collects data on Amsterdam inhabitants, its housing stock, employment, and entrepreneurial activities in the city. The characteristics of the panel members enable us to compare three social groups with similar levels of education but different migration experiences. The survey was designed for the migration, transformation, and sustainability (MISTY) project that explored the ways that migration interacts with sustainability concerns in cities of destination.^4^ The survey included a module on the impacts of the COVID-19 crisis on respondents’ health, social contacts, and income in the household. Among others, respondents were asked to compare these three aspects of their lives before and after the pandemic started. The online data collection took place between 14 and 28th July 2020, and the response rate was 49%.

### Sample Characteristics

A total of 1383 individuals participated in our survey. After elimination of missing observations, we end up with a final dataset of 1056 individuals, of which 614 (58%) were non-migrants, 179 (17%) were international migrants, and 264 (25%) were second-generation residents.^5^ These three groups in our sample have, on average, relatively comparable socio-demographic characteristics (see Table 2). The decision to parse out migrants into two generations reflects that those born within the Netherlands share a different level of embeddedness than groups who immigrated to the country. This also aligns our findings with the 2020 statistical definitions for migrant groups in the Netherlands.

**Table 2:** Sample characteristics

Variables		Non-migrants (%)	International migrants (%)	Second-generation residents (%)	Total sample (%)
*Individual and household characteristics*					
Age (mean)		58.09	55.36	54.51	56.65
Gender	Male Female	55.70	50.84	50.38	53.55
		44.30	49.16	49.62	46.45
Education	None/primary	11.73	15.64	13.64	12.87
	Secondary	8.14	8.94	14.77	9.93
	Bachelor	33.06	29.05	29.55	31.5
	Master	47.07	46.37	42.05	45.7
Position in labor market	Paid employment	44.95	44.13	50.00	46.07
	Self-employed	17.26	18.99	15.91	17.22
	Unemployed	5.86	10.06	7.95	7.1
	Retired	30.13	24.58	25.00	27.91
	Other	1.79	2.23	1.14	1.7
Household	Single person	36.32	43.58	35.61	37.37
composition	Couple without kids	40.39	29.05	37.5	37.75
	Couple with kids	18.4	16.76	19.7	18.45
	Single parent with kids	3.26	6.7	4.92	4.26
	Other	1.63	3.91	2.27	2.18
*Local embeddedness*					
Years in Amsterdam (mean)		38.45	32.22	36.27	36.85
Relative wealth (mean)		3.30	3.23	3.21	3.27
Social contact with neighbors		3.43	3.39	3.39	3.41
Feel at home in neighborhood		3.63	3.49	3.60	3.60
*Dependent variables – impact of the pandemic*					
Lower income	No	79.87	71.82	79.03	78.29
	Yes	20.13	28.18	20.97	21.71
Fewer social contracts	No	60.55	49.72	54.31	57.14
	Yes	39.45	50.28	45.69	42.86
Worse health	No	85.06	82.87	80.15	83.46
	Yes	14.94	17.13	19.85	16.54

The average age of respondents in our sample was 57 years. This relatively higher age group is probably related to the context in which our survey took place. The start of the COVID-19 pandemic was an insecure time, in which many individuals and families were struggling to balance work and family life. As such, we probably reached individuals who had relatively more time and energy to respond to our survey. As the table shows, we also have a relatively high proportion of respondents who are retired. Although it is clear that our sample is not representative of the Amsterdam population, the data is still suitable for the purpose of our study: to investigate how local embeddedness affect the impact of the COVID-19 pandemic on respondents’ lives.

There are slightly more males in our sample (54%) than females (46%) and the majority of respondents (46%) is highly educated. The differences in education levels between the three groups are not large, although international migrants seem to be slightly lower educated on average. For example, 16% of international respondents had none or primary education, as compared to 12% of non-migrants and 14% of second-generation residents. In terms of household composition, we can see that there are more single individuals without children among the international migrants, and that couples without kids are overrepresented among non-migrants. Overall, most respondents are either single without kids, or part of a couple without kids.

### Measuring Local Embeddedness

The three dimensions of local embeddedness include economic, social network, and psychosocial embeddedness (Ruben et al., 2009). Economic embeddedness, which relates to tangible and material aspects such as employment or asset ownership, is operationalized here using a variable measuring relative wealth. Relative wealth is measured with the question “*Compared to your neighbors, how would you describe your financial situation?*” with answer categories ranging from (1) one of the poorest to (5) one of the richest. The average score among respondents is 3.27, which is in between the “about average” and “above average” answer categories. The non-migrants in our sample score slightly higher on the relative wealth variable than international migrants and second-generation residents, but the difference is not statistically significant.

We operationalize social embeddedness, defined as the social relations and information that an individual has access to, using social contacts with neighbors. This variable was measured with the statement “I have a lot of contact with my neighbors,” with answer categories ranging from (1) totally disagree to (5) totally agree. The average response on social contacts with neighbors across all respondents is 3.41, and the differences between respondents with different migration experiences are not statistically significant.

Finally, psychosocial embeddedness is operationalized as “feeling at home,” measured with the statement “I feel at home with the people who live in this neighborhood.” Again, answer categories range from (1) totally disagree to (5) totally agree. The average response to this statement was 3.60, and although international migrants score slightly lower on the feeling at home variable, the difference is not statistically significant.

In addition to economic, social, and psychosocial embeddedness, we include time of residence in Amsterdam, and we hypothesize that more time in Amsterdam is likely to increase respondents’ local embeddedness. We therefore created a variable accounting for the years of residence since respondents moved to Amsterdam for the last time. The average time that the international migrants in our sample spent in the city is around 32 years, which is about 4 years less than the value for second-generation residents and 6 years less than the value for non-migrants. This reflects that our sample includes internal migrants and Amsterdam born returned migrants—some of which might not necessarily be more locally embedded than some international migrants.

### Measuring Vulnerability

To measure vulnerability, we apply an asset vulnerability framework designed for urban areas (Moser, 1998) and focus on three key assets that could have been affected by the pandemic: economic, social, and human capital (health). Underlying the asset vulnerability framework lies the assumption that individuals or households that have relatively more assets or a diverse portfolio of assets are more resilient and less vulnerable to gradually changing circumstances or shocks. Research has shown that asset frameworks are a useful tool for studying vulnerability during or after disasters as they provide a comprehensive and multi-dimensional overview of the challenges faced by different groups, which can inform targeting and design of policies and programs to support those who are most vulnerable (Zhang et al., 2020).

The original asset vulnerability framework contains five asset categories, including financial, human, natural, physical, and social capital. Rooted in the more classic poverty measurement approaches and livelihood approaches, vulnerability approaches present a dynamic picture of whether an individual or household is at risk of falling into poverty (Zhang et al., 2020). As vulnerability measurements need to be relevant for the context in which they are conducted (Birkmann, 2006; Birkmann & Wisner, 2006; Wisner et al., 2003), we focus on impacts that are most related to the current pandemic, including impacts on economic, social, and human (health) capital.

To measure vulnerability, we asked respondents to indicate whether their economic lives, social lives, or health deteriorated or improved due to the corona crisis. Respondents could indicate whether their lives deteriorated very much, deteriorated, stayed the same, improved, or improved very much. These responses were recoded into a variable that measured whether individuals experienced a deterioration or not (0 = no, 1 = yes), clustering respondents who reported “very much deteriorated” or “deteriorated” as those who were negatively affected.

### Measuring Dependency on the Labor Market

We use the position in the labor market as an indicator of respondents’ degree of dependency on the market for their livelihoods, with the assumption that those who are in paid employment are less vulnerable to economic shocks than the self-employed, but are more vulnerable than retirees, who do not rely on the labor market as main source of income. Self-employment figures have risen sharply in the Netherlands over the past years. In Amsterdam, 17.1% of the population is self-employed, which is significantly higher than the national average (CBS 2019). While acknowledging that self-employed individuals can also be strongly embedded in their local environment, and that paid employment might also be temporary, research has shown that self-employed individuals are generally more vulnerable during economic downturns. The Central Bureau of Statistics has calculated that more than 50% of self-employed individuals witnessed a decrease in the demand for their services during the pandemic (CBS 2021). This is particularly the case for self-employed individuals who work in the sectors which are hit hard by the pandemic, such as construction, transportation (e.g., taxi drivers), retail, and catering. We therefore distinguish between paid employment, self-employment, unemployed, retired, and other (e.g., students). In our sample, 46% is in paid employment, 17% is self-employed, and 7% is unemployed. As some individuals may have lost their jobs due to the pandemic, we ran robustness checks excluding those respondents who responded positively to a question on whether someone in their household had lost their job due to the pandemic and those who were unemployed (21 individuals in total). The results (available upon request) did not change when these individuals were excluded. As described earlier, we have a relatively high proportion of retired individuals in our sample (28%). Occupational status is comparable across our three groups, although second-generation residents are more likely to be in paid employment, whereas the international migrants are slightly more likely to be self-employed or unemployed.

### Analyses

In the following sections, we run descriptive statistics and logistic regression analyses to explore how local embeddedness is related to the three dimensions of vulnerability: economic, social, and health-related vulnerability. In the regression analyses, we introduce the independent variables in a step-wise manner to check the robustness of the results. In the first model, we introduce the individual and household variables, whereas in the second model, we add the variables related to economic, social, and psychosocial embeddedness. Finally, in the third model, we control for vulnerability to the pandemic in the other subdimensions. For example, when we present the analyses on economic vulnerability, we control for social and health-related vulnerability, to account for the fact that multiple vulnerabilities may interact and overlap. We run three sets of regression analyses. The first two sets included age and years of residence in Amsterdam respectively to overcome the observed multicollinearity when we included the two variables in the same models. In the third set, we run the analyses separately by migration status sub-group, to explore whether the impact of local embeddedness on vulnerability differs for the non-migrants, international migrants and second-generation residents in our sample. The number of observations for international migrants and second-generation residents are relatively small, and should therefore be treated with caution, although results seem robust. The analyses are controlled for the neighborhood in which the participant resided at the time of the survey, to account for neighborhood effects (results are available upon request). In the following pages, we present the results of the first set of analyses with age. The analyses comparing the different migrant groups are available in the Appendix.

## Findings: Vulnerabilities to the COVID-19 Pandemic

**Fig. 3:**
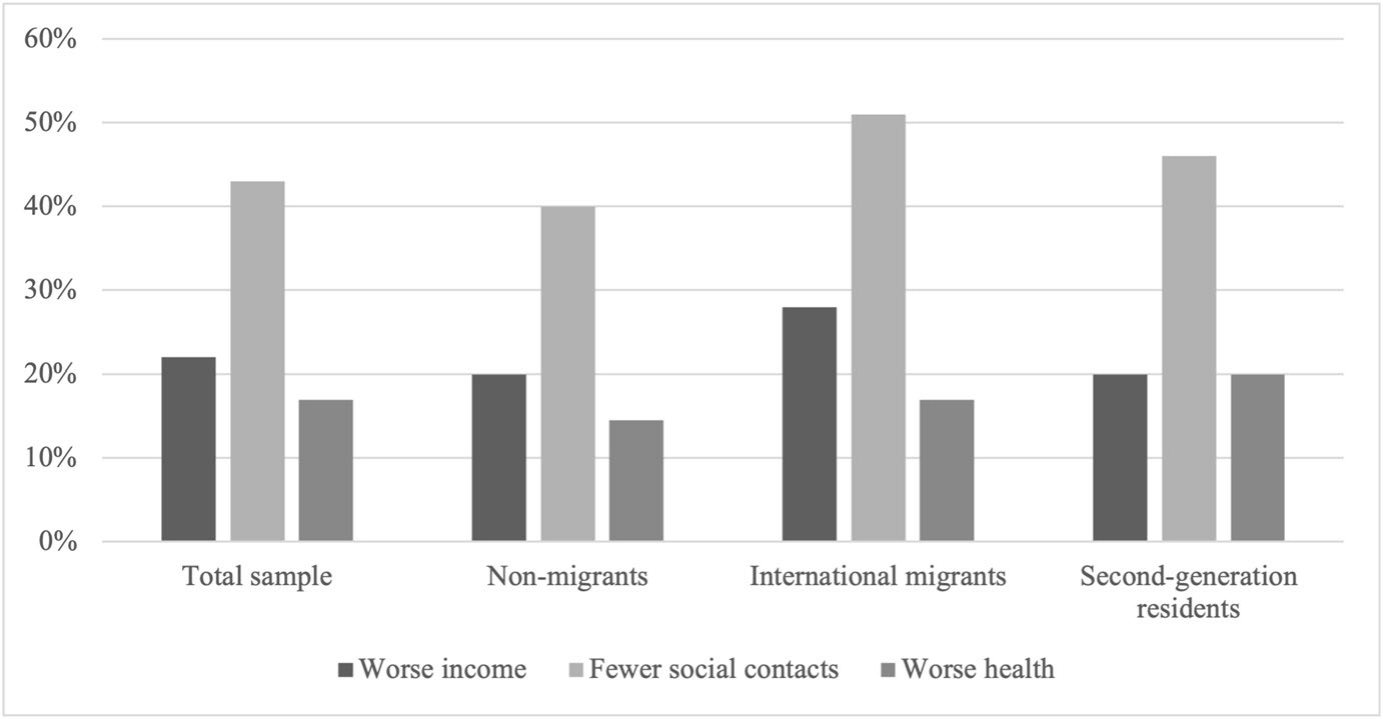
Impacts of the COVID-19 pandemic on respondents

Initial observations of the three groups in our sample show high levels of vulnerability to the pandemic across all three groups (Fig. 3). Forty-three percent of respondents in our sample reported how they had experienced a decrease in their social contacts, 22% reported a decrease in their income, and 17% reported worse health outcomes after the start of the pandemic. International migrants were more vulnerable than non-migrants on all indicators, and particularly in the social dimension. Approximately half (51%) of the international migrants reported fewer social contacts, 28% reported worse income, and 17% witnessed a deterioration of health. Second-generation resident also reported a higher impact on their social lives than non-migrants, but seemed to be particularly vulnerable in the health dimension as compared to non-migrants and international migrants. These findings align with previous studies conducted in Amsterdam, described above, which showed that individuals born abroad or with at least one parent born abroad were more likely to lose their jobs due to the pandemic (Atlas voor Gemeenten 2020) and were more likely to catch COVID-19 and to be hospitalized than other city residents (GGD Amsterdam, 2021a, b). In the following sections, we will explore these descriptive findings in more detail by running regression analyses and controlling for other individual- and household-level factors, including the level of embeddedness that individuals experience in the city, that might play a role in the vulnerability outcomes of our respondents.

### Vulnerability to Lost Income During the Pandemic

Table 3 shows the results of the logistic regression analysis using lost household income during the pandemic (yes/no) as the dependent variable. In terms of individual- and household-level factors, the results reveal that older individuals were less vulnerable to the economic impacts of the pandemic. This finding is in line with a previous study that found that young people were more likely to lose their job in Amsterdam during the pandemic (Atlas voor Gemeenten 2020).

**Table 3:** Household income loss during the pandemic: regression analyses

		(1)	(2)	(3)
Age (ref. ≤ 35)	Age (35–49)	− 0.36	− 0.78**	− 0.80**
		(0.30)	(0.35)	(0.35)
	Age (50–64)	− 0.26	− 0.82**	− 0.77**
		(0.27)	(0.33)	(0.33)
	Age (65 >)	− 1.11***	− 1.16***	− 1.11**
		(0.30)	(0.44)	(0.44)
Gender (ref. = female)	Male	0.33**	0.30	0.31
		(0.16)	(0.19)	(0.19)
Education (ref. = no/primary)	Secondary	0.47	0.33	0.26
		(0.34)	(0.40)	(0.40)
	Bachelor	0.28	0.29	0.24
		(0.28)	(0.32)	(0.33)
	Master or higher	0.32	0.46	0.39
		(0.28)	(0.33)	(0.33)
Migrant background (ref. = non-migrant)	International migrants	0.43**	0.42*	0.40*
		(0.20)	(0.24)	(0.24)
	Second-gen. residents	− 0.01	0.02	− 0.01
		(0.19)	(0.22)	(0.22)
Household composition (ref. = single, no children)	Couple (no children)	− 0.23	− 0.08	− 0.08
		(0.19)	(0.23)	(0.23)
	Couple (children)	0.19	0.44*	0.48*
		(0.22)	(0.27)	(0.27)
	Single parent (children)	0.68*	0.48	0.46
		(0.35)	(0.42)	(0.43)
	Other	0.47	0.31	0.42
		(0.48)	(0.60)	(0.59)
Relative wealth			− 0.63***	− 0.62***
			(0.11)	(0.11)
Occupational status (ref. = retired)	In paid employment		0.14	0.15
			(0.40)	(0.41)
	Self-employed/freelance		2.69***	2.74***
			(0.37)	(0.37)
	Unemployed		1.47***	1.37***
			(0.46)	(0.46)
	Other		0.68	0.53
			(0.71)	(0.72)
Contact with neighbors			0.21**	0.21**
			(0.10)	(0.10)
Feeling at home			− 0.21*	− 0.19
			(0.12)	(0.12)
Decreased social contacts				0.28
				(0.19)
Worse health				0.45*
				(0.24)
Constant		− 1.17***	− 0.26	− 0.57
		(0.43)	(0.75)	(0.75)
Observations		1,063	1,063	1,063
Pseudo R2		0.05	0.26	0.27

Standard errors in parentheses. *** *p* < 0.01, ** *p* < 0.05, * *p* < 0.1. Analyses are controlled for neighborhood effects

Several significant findings for the individual- and household-level factors in our model become insignificant when controlling for the degree of dependency on the labor market, which shows that the position in the labor market is an important variable that mediates the impact of the pandemic for different socio-demographic groups. For example, male respondents were more likely to report income loss, which relates to the fact that males in our sample were more often self-employed than female respondents. Furthermore, those who were retired were less vulnerable to lost income than the other categories, and those in paid employment less vulnerable than the self-employed and the unemployed. As some individuals may have lost their jobs *during* the pandemic and were therefore unemployed because of the pandemic, we re-ran the analyses excluding those individuals. The results did not change, which suggests that the results are robust.

International migrants were more likely to experience income loss during the pandemic, and in terms of household composition, couples with children were more vulnerable to income loss. This could be due to several reasons. First, individuals in households with children were more likely to be self-employed than individuals in other households: 22% of households with children reported self-employment as the main income source, whereas this was 15% for single households and 17% couples without kids. Second, 13% of households with children reported job loss during the pandemic, versus 10 and 9% of households without kids and single households, respectively. Moreover, it is likely that households with children faced additional constraints to their employment situation due to the closure of schools and daycare.

Variables related to the embeddedness of individuals (models 2 and 3) show how economic embeddedness (relative wealth) is negatively related to the economic impact of the pandemic. Those with higher self-reported relative wealth were less likely to experience income loss due to the pandemic. In terms of social embeddedness, the results reveal that respondents with more social contacts with their neighbors had a higher likelihood of income loss. This is probably a case of reversed causality, in the sense that those who experienced income loss became more reliant on their social contacts. Those who felt more at home in their neighborhoods were less likely to experience an income loss during the pandemic, although this variable is no longer significant when we control for a decrease in social contacts. Yet, the finding suggests that higher psychosocial embeddedness in the neighborhood makes individuals less vulnerable to economic impacts of the pandemic. When replacing the variable age with time spent in the city (results available upon request), we see that the longer respondents are in the city, the less vulnerable they are to household income loss due to the pandemic.

Finally, the different vulnerabilities that respondents in our sample experienced during the pandemic are related. Those who reported worse health outcomes due to the pandemic were more likely to experience income loss. Whether worse health outcomes are the result of income loss, or the other way around, cannot be deducted from these findings, but the relationship could work both ways. A decrease in social contacts due to the pandemic was not significantly related to a loss of household income.

Table 6 in the Appendix displays the results for the economic vulnerability dimension split by migration background. Overall, the findings indicate that economic embeddedness plays a similar role for non-migrants, international migrants, and second-generation residents. Those with higher relative wealth are less vulnerable in economic terms. In terms of social and psychosocial embeddedness, the results are more mixed. Contacts with neighbors are important for the international migrants in our sample—having a lot of contact with neighbors is positively related to their vulnerability to lost income during the pandemic, which could indicate that they are more dependent on social networks to secure their livelihoods. Subsequently, having less opportunities of social contact during the pandemic could have reduced their income. Feeling at home in the neighborhood plays a significant role for the economic vulnerability of non-migrants and second-generation residents—it reduces their vulnerability to income loss. Feeling at home could be correlated with housing ownership or the length of time since the rental agreement. Non-migrants and second-generation residents are more likely to own their house, and pay lower mortgages and rents than newcomers who arrived to Amsterdam in a period with higher prices in the housing market. The fact that newcomers are more economically vulnerable than old migrants reinforces this idea. Self-employment and unemployment remain important determining factors for economic vulnerability during the pandemic for all groups: those who were self-employed or unemployed were more likely to report income loss due to the pandemic, regardless of their migration background.

### Social Vulnerability due to the Pandemic

We now turn our attention to our second outcome variable, social vulnerability, which describes whether respondents experienced a decrease in their social contacts during the pandemic (Table 4). In terms of individual- and household-level characteristics, the findings show how those who are higher educated were more likely to experience a decrease in their social contacts. Higher educated respondents in our sample and particularly those with a master’s degree were younger on average than other respondents, and had spent less time in the city, which made them socially less embedded and therefore more vulnerable to a reduction in their social contacts. They were also less likely than other respondents to be born in Amsterdam. Likewise, international migrants were also more socially vulnerable due to the pandemic. Having spent less time in the city and being less embedded in their neighborhoods, international migrants were more likely to experience a decrease in their social contacts during the pandemic. Single parents were also more likely to report a decrease in their social contacts, which is most likely due to the dual strain of parenting and work during the pandemic.

**Table 4:** Decreased social contacts during the pandemic: regression analyses

		(1)	(2)	(3)
Age (ref. ≤ 35)	Age (35–49)	− 0.00	0.10	0.14
		(0.27)	(0.27)	(0.28)
	Age (50–64)	− 0.47*	− 0.39	− 0.29
		(0.25)	(0.26)	(0.27)
	Age (65 >)	− 0.68***	− 0.62*	− 0.47
		(0.26)	(0.34)	(0.35)
Gender (ref. = female)	Male	− 0.05	− 0.05	− 0.07
		(0.13)	(0.13)	(0.14)
Education (ref. = no/primary)	Secondary	0.61**	0.65**	0.59**
		(0.28)	(0.28)	(0.29)
	Bachelor	0.33	0.41*	0.36
		(0.22)	(0.23)	(0.23)
	Master or higher	0.61***	0.79***	0.74***
		(0.22)	(0.23)	(0.23)
Migrant background (ref. = non-migrant)	International migrants	0.47***	0.46**	0.46**
		(0.18)	(0.18)	(0.18)
	Second-gen. Residents	0.23	0.24	0.21
		(0.15)	(0.16)	(0.16)
Household composition (ref. = single, no children)	Single (parent)	0.32**	0.40***	0.45***
		(0.15)	(0.16)	(0.16)
	Couple (no children)	− 0.21	− 0.07	− 0.03
		(0.20)	(0.20)	(0.21)
	Couple (parents)	− 0.02	− 0.10	− 0.16
		(0.33)	(0.34)	(0.35)
	Other	0.24	0.03	0.21
		(0.46)	(0.47)	(0.48)
Relative wealth			− 0.18**	− 0.15*
			(0.08)	(0.08)
Occupational status (ref. = retired)	In paid employment		− 0.14	− 0.09
			(0.28)	(0.28)
	Self-employed/freelance		0.02	0.00
			(0.28)	(0.29)
	Unemployed		0.43	0.19
			(0.36)	(0.37)
	Other		0.29	− 0.07
			(0.58)	(0.61)
Contact with neighbors			− 0.01	− 0.02
			(0.07)	(0.08)
Feeling at home			− 0.19**	− 0.16*
			(0.09)	(0.09)
Worse income				0.24
				(0.19)
Worse health				1.16***
				(0.19)
Constant		− 0.72***	0.36	− 0.19
		(0.36)	(0.55)	(0.57)
Observations		1,063	1,063	1,063
Pseudo R2		0.04	0.05	0.08

Standard errors in parentheses. *** *p* < 0.01, ** *p* < 0.05, * *p* < 0.1. Analyses are controlled for neighborhood effects

The economic embeddedness variables show that those with lower self-reported relative wealth were socially more vulnerable. However, whereas position in the labor market was an important factor impacting the economic impact of the pandemic, economic embeddedness has no direct impact on the social vulnerability of our respondents. Social embeddedness, measured by contacts with neighbors, is also not significantly related to decreased social contacts during the pandemic, but psychosocial embeddedness is important: those who reported to feel more at home in the neighborhood were less likely to report a decrease in their social contacts during the pandemic. The findings in Table 4 also highlight again that the different vulnerabilities are related, as respondents who reported worse health outcomes were also more likely to experience a decrease in their social contacts. These findings show how the different vulnerabilities (economic, social, and health) overlap and interact.

Table 7 in the Appendix shows the analyses for social vulnerability split by the migration status of our respondents. Among international migrants and non-migrants, single parents are particularly vulnerable to decreased social contacts, whereas single second-generation residents with children are not particularly affected. Single international migrants with children might lack the family or social support to take care of their children during the pandemic. Additionally, second-generation single parents could have more informal support than non-migrants, which could be explained by cultural factors not included in our model. The analysis also reveals links between social and health vulnerabilities for the three groups.

### Vulnerability to Worse Health Outcomes During the Pandemic

Our final outcome of interest is whether respondents reported worse health outcomes since the pandemic’s onset, with the results shown in Table 5. As compared to the results for economic and social vulnerability, there are few statistically significant findings. For example, characteristics such as age, time, gender, education level, and migrant background do not seem to be significantly related to health outcomes, when controlling for other factors. Whereas the descriptive statistics showed that second-generation residents were more likely to report worse health outcomes, this effect is no longer significant when controlling for education level and relative wealth. This finding suggests that worse health outcomes are not related to migration background per se, but are mediated by other socio-economic factors.

**Table 5:** Worse health outcomes during the pandemic: regression analyses

		(1)	(2)	(3)
Age (ref. ≤ 35)	Age (35–49)	− 0.22	0.05	0.09
		(0.31)	(0.33)	(0.34)
	Age (50–64)	− 0.69**	− 0.54*	− 0.39
		(0.29)	(0.32)	(0.33)
	Age (65 >)	− 0.96***	− 0.88*	− 0.70
		(0.31)	(0.47)	(0.49)
Gender (ref. = female)	Male	0.00	0.05	0.03
		(0.17)	(0.18)	(0.18)
Education (ref. = no/primary)	Secondary	0.47	0.51	0.35
		(0.36)	(0.38)	(0.39)
	Bachelor	0.25	0.42	0.30
		(0.30)	(0.31)	(0.32)
	Master or higher	0.13	0.49	0.25
		(0.30)	(0.32)	(0.33)
Migrant background (ref. = non-migrant)	First-generation	0.10	0.07	− 0.08
		(0.24)	(0.24)	(0.25)
	Second-generation	0.24	0.29	0.19
		(0.20)	(0.20)	(0.21)
Household composition (ref. = single, no children)	Single (parent)	− 0.29	− 0.20	− 0.27
		(0.20)	(0.21)	(0.21)
	Couple (no children)	− 0.52**	− 0.35	− 0.37
		(0.26)	(0.27)	(0.28)
	Couple (parents)	0.23	0.22	0.25
		(0.39)	(0.40)	(0.42)
	Other	− 0.76	− 1.18*	− 1.25*
		(0.65)	(0.69)	(0.71)
Relative wealth			− 0.13	− 0.05
			(0.10)	(0.11)
Occupational status (ref. = retired)	In paid employment		− 0.34	− 0.35
			(0.40)	(0.43)
	Self-employed/freelance		− 0.72*	− 1.05**
			(0.43)	(0.47)
	Unemployed		0.92**	0.72
			(0.46)	(0.48)
	Other		1.41**	1.34**
			(0.64)	(0.67)
Contact with neighbors			0.03	0.04
			(0.10)	(0.10)
Feeling at home			− 0.23**	− 0.18
			(0.11)	(0.11)
Worse income				0.48**
				(0.23)
Decreased social contacts				1.15***
				(0.19)
Constant		− 0.88*	− 0.01	− 0.88
		(0.45)	(0.73)	(0.77)
Observations		1,063	1,063	1,063
Pseudo R2		0.04	0.08	0.13

Standard errors in parentheses. *** *p* < 0.01, ** *p* < 0.05, * *p* < 0.1. Analyses are controlled for neighborhood effects

Position in the labor market does seem to play an important role in health outcomes. Those who were self-employed were significantly less vulnerable to worse health outcomes than those who were in paid employment, whereas those who were unemployed were significantly and negatively impacted in terms of their health. It is likely that these individuals experienced negative health outcomes, which, in some cases, may have led to their unemployment. Indeed, the data reveals that those with worse health outcomes more often reported that they had lost their job due to COVID-19. Economic, social, and psychosocial embeddedness played less of a role in deteriorated health outcomes as a result of the pandemic. Finally, worse income due to the pandemic and a reduction in social contacts are significantly related to worse health outcomes.

Finally, results for the health vulnerability dimension split by migration background (Table 8 in the Appendix) show that social vulnerability during the pandemic significantly increased health vulnerability in the three groups, particularly for international migrants and second-generation residents. This suggests that more isolated respondents during the pandemic could have been more vulnerable to mental health problems, or that health issues due to the pandemic led to social isolation. In terms of embeddedness, we can observe that those who reported to “feel at home” were less likely to report negative health outcomes for particularly non-migrants and second-generation residents in the sample, whereas social embeddedness made second-generation residents less vulnerable in terms of health as well.

## Discussion and Conclusions

This paper studied the economic, social, and health-related vulnerabilities to the COVID-19 pandemic of residents of the city of Amsterdam, with a particular focus on the role of local embeddedness as a factor that influences the impacts of the pandemic. We hypothesized that those who were more locally embedded, in economic, social, and psychosocial terms (Ruben et al., 2009), were less likely to experience negative (side)effects of the pandemic, including a loss in income, reduced social contacts, or worse health outcomes. Our data were collected in July 2020, 3 to 4 months after the start of the pandemic, and therefore provide insights into its early impacts. Our sample included individuals with diverse migration backgrounds, but relatively similar socio-demographic profiles, which allowed us to study how local embeddedness affected the extent to which individuals were impacted by the pandemic. We argued that the concept of local embeddedness is applicable to individuals with different migration backgrounds (non-migrants, international migrants, and second-generation residents), as individuals spent different periods in the city, had different positions in the labor market, and varying levels of social contacts.

Our descriptive statistics show that the COVID-19 pandemic had a large impact on the respondents in our sample, particularly in terms of their social contacts. Forty-three percent of respondents in our sample reported a decrease in their social contacts, 22% reported a decrease in their income, and 17% reported worse health outcomes after the start of the pandemic. In line with previous studies conducted in Amsterdam (Atlas voor Gemeenten 2020) and elsewhere (Guadagno, 2020), international migrants and second-generation residents were most vulnerable to the negative impacts of the pandemic. However, whereas international migrants in our sample were more likely to report a decrease in their social contacts, second-generation residents had worse health outcomes. A previous study has shown that worse health outcomes among second-generation residents is a result of a combination of vulnerability factors, such as low socio-economic status, underlying health conditions, living conditions, and working conditions, as well as language difficulties (GGD Amsterdam, 2021a, b). In assessing these factors, statistical organizations at the municipal and national level should continue strengthening their capacity to specifically gather data about migrant populations and identify the unique challenges they face depending on their varying levels of embeddedness.

However, confirming our hypothesis, those who were more embedded in the city of Amsterdam and/or their neighborhoods were less vulnerable during the COVID-19 pandemic, regardless of their migration background. Nevertheless, the relations between the three dimensions of embeddedness and our vulnerability outcomes differed across the types of vulnerability we included in our analyses. Economic, social, and psychosocial embeddedness and time of residence played a particularly important role in moderating the economic and social impacts of the COVID-19 pandemic, and less of a role for the health impact of the pandemic. For example, those with higher relative wealth before the pandemic were less vulnerable economically and socially, and individuals who had more social contacts and who felt more at home before the pandemic experienced less of a reduction in their social contacts. Health outcomes were mostly affected by employment status, and we find that higher vulnerability in health outcomes among second-generation residents is related to other socio-economic factors such as education, relative wealth, and neighborhood effects. Among the lessons learned is that residents’ likelihood of being negatively affected by the pandemic could be reduced via targeted interventions. For example, initiatives supporting secure employment, strong social relations, well-disseminated information, and accessibility to social security programs for all, including people with zero contract hours and those who are self-employed, are likely to foster communities that are less-severely affected by shocks such as a pandemic.

Our study shows that although international migrants are particularly vulnerable to the COVID-19 pandemic, studies should take into account their degree of embeddedness in their place of residence and distinguish between different cohorts of migrants—newcomers versus older generations of migrants. Our findings also show how different vulnerabilities overlap and interact, as those who reported worse health outcomes, for example, were also more vulnerable in terms of their income and social contacts. Policies and programs that are implemented to mitigate the impacts of the pandemic on city residents should, therefore, take into account this multidimensional nature of vulnerability. As such, our findings unveil the close interplay between economy and society in shaping people’s vulnerabilities during the COVID-19 pandemic.

## Data Availability

The data that support the findings of this study are available from the corresponding author upon reasonable request.

## Appendix

Map1

Table

**Table 6:** Household income loss during the pandemic: regression analyses by migration status

		Non-migrants	International migrants	Second-generation residents
Age (ref. ≤ 35)	Age (35–49)	− 0.60	− 2.02**	− 0.24
		(0.53)	(0.89)	(0.73)
	Age (50–64)	− 0.68	− 1.49*	− 0.16
		(0.49)	(0.89)	(0.73)
	Age (65 >)	− 0.79	− 2.02*	− 1.73
		(0.58)	(1.22)	(1.16)
Gender (ref. = female)	Male	0.44*	0.19	0.07
		(0.26)	(0.52)	(0.44)
Education (ref. = no/primary)	Secondary	0.15	0.90	0.12
		(0.57)	(1.17)	(0.80)
	Bachelor	0.18	0.79	− 0.36
		(0.45)	(0.78)	(0.73)
	Master or higher	0.27	0.50	0.11
		(0.45)	(0.88)	(0.73)
Household composition (ref. = single, no children)	Couple (no children)	− 0.49	− 0.34	1.59***
		(0.30)	(0.68)	(0.60)
	Couple (children)	0.43	0.18	1.38**
		(0.36)	(0.73)	(0.69)
	Single parent (children)	1.05*	0.14	0.19
		(0.60)	(1.05)	(1.13)
	Other	0.34	− 0.02	1.49
		(0.88)	(1.24)	(1.41)
Relative wealth		− 0.57***	− 1.05***	− 1.02***
		(0.14)	(0.32)	(0.27)
Occupational status (ref. = retired)	Paid employment	0.16	0.17	− 0.04
		(0.51)	(1.22)	(1.09)
	Self-employed/freelance	2.57***	3.20***	3.86***
		(0.46)	(1.16)	(1.06)
	Unemployed	1.26**	2.22*	0.89
		(0.61)	(1.34)	(1.16)
	Other	0.53	1.23	− 0.01
		(0.87)	(2.12)	(1.86)
Contact with neighbors		0.17	0.57*	0.07
		(0.14)	(0.31)	(0.24)
Feeling at home		− 0.30*	0.11	− 0.52*
		(0.16)	(0.31)	(0.29)
Decreased social contacts		0.08	1.29**	0.58
		(0.25)	(0.53)	(0.48)
Worse health		0.63**	0.43	0.01
		(0.32)	(0.68)	(0.55)
Constant		0.05	− 1.67	1.24
		(1.02)	(1.91)	(1.86)
Observations		616	180	267
Pseudo R2		0.25	0.37	0.27

Standard errors in parentheses. *** *p* < 0.01, ** *p* < 0.05, * *p* < 0.1. Analyses are controlled for neighborhood effects

6

Table

**Table 7:** Decreased social contacts during the pandemic: regression analyses by migration status

		Non-migrants	International migrants	Second-generation residents
Age (ref. ≤ 35)	Age (35–49)	0.35	0.56	0.03
		(0.40)	(0.88)	(0.53)
	Age (50–64)	− 0.08	− 0.28	− 0.41
		(0.37)	(0.84)	(0.52)
	Age (65 >)	− 0.33	− 1.68	− 0.20
		(0.46)	(1.12)	(0.82)
Gender (ref. = female)	Male	0.06	− 0.82*	− 0.03
		(0.18)	(0.42)	(0.29)
Education (ref. = no/primary)	Secondary	0.58	− 0.35	0.90*
		(0.42)	(0.84)	(0.54)
	Bachelor	0.22	0.31	0.60
		(0.32)	(0.62)	(0.48)
	Master or higher	0.64**	0.84	1.06**
		(0.32)	(0.66)	(0.49)
Household composition (ref. = single, no children)	Single (parent)	0.44**	1.85***	− 0.15
		(0.21)	(0.54)	(0.34)
	Couple (no children)	0.20	− 0.01	− 0.65
		(0.28)	(0.59)	(0.45)
	Couple (parents)	− 0.43	− 0.59	0.08
		(0.54)	(0.83)	(0.73)
	Other	0.33	− 0.21	0.24
		(0.70)	(1.24)	(0.97)
Relative wealth		− 0.24**	0.06	− 0.13
		(0.11)	(0.23)	(0.19)
Occupational status (ref. = paid employment)	Self-Employed	− 0.15	− 1.46	0.03
		(0.35)	(0.96)	(0.72)
	Unemployed	0.10	− 0.96	− 0.60
		(0.36)	(0.92)	(0.77)
	Retired	0.10	− 1.44	0.97
		(0.49)	(1.15)	(0.87)
	Other	− 0.45	-	− 0.28
		(0.73)		(1.71)
Contact with neighbors		0.03	0.05	− 0.16
		(0.10)	(0.24)	(0.16)
Feeling at home		− 0.18	− 0.05	− 0.26
		(0.12)	(0.24)	(0.19)
Worse income		0.02	1.09**	0.39
		(0.25)	(0.50)	(0.46)
Worse health		0.98***	1.56***	1.53***
		(0.25)	(0.58)	(0.40)
Constant		0.07	− 0.59	0.62
		(0.76)	(1.58)	(1.26)
Observations		616	177	267
Pseudo R2		0.06	0.23	0.15

Standard errors in parentheses. *** *p* < 0.01, ** *p* < 0.05, * *p* < 0.1. Analyses are controlled for neighborhood effects

7

Table

**Table 8:** Worse health outcomes during the pandemic: regression analyses by migration status

		Non-migrants	International migrants	Second-generation residents
Age (ref. ≤ 35)	Age (35–49)	− 0.08	0.58	0.13
		(0.53)	(0.95)	(0.64)
	Age (50–64)	− 0.23	− 0.07	− 1.04
		(0.49)	(0.95)	(0.70)
	Age (65 >)	− 1.03	− 1.11	0.27
		(0.64)	(2.51)	(1.16)
Gender (ref. = female)	Male	0.05	− 0.92	0.40
		(0.26)	(0.58)	(0.40)
Education (ref. = no/primary)	Secondary	0.60	− 0.76	− 0.03
		(0.60)	(1.55)	(0.68)
	Bachelor	0.72	0.57	− 0.26
		(0.51)	(0.98)	(0.61)
	Master or higher	0.37	1.84*	0.14
		(0.52)	(1.03)	(0.64)
Household composition (ref. = single, no children)	Single (parent)	− 0.25	− 1.54*	− 0.21
		(0.29)	(0.80)	(0.47)
	Couple (no children)	− 0.51	− 0.06	0.28
		(0.40)	(0.81)	(0.59)
	Couple (parents)	− 1.25	1.19	1.36
		(1.08)	(0.99)	(0.93)
	Other	− 1.27	− 1.35	− 1.77
		(1.14)	(1.57)	(1.32)
Relative wealth		0.21	− 0.59*	− 0.47**
		(0.15)	(0.31)	(0.24)
Occupational status (ref. = paid employment)	Self-Employed	− 0.62	− 1.44	− 0.03
		(0.52)	(2.46)	(1.07)
	Unemployed	− 1.21**	− 3.31	− 0.28
		(0.57)	(2.54)	(1.17)
	Retired	0.25	− 0.83	2.17*
		(0.64)	(2.54)	(1.11)
	Other	1.76**	0.16	1.12
		(0.80)	(3.13)	(2.75)
Contact with neighbors		0.23	0.21	− 0.56**
		(0.15)	(0.32)	(0.23)
Feeling at home		− 0.39**	− 0.41	0.48*
		(0.16)	(0.35)	(0.25)
Worse income		0.70**	0.30	0.23
		(0.32)	(0.70)	(0.54)
Decreased social contacts		0.98***	1.72***	1.62***
		(0.25)	(0.64)	(0.42)
Constant		− 1.83*	1.97	− 0.70
		(1.09)	(2.99)	(1.72)
Observations		616	180	267
Pseudo R2		0.13	0.33	0.28

Standard errors in parentheses. *** *p* < 0.01, ** *p* < 0.05, * *p* < 0.1. Analyses are controlled for neighborhood effects

8

## Footnotes

1 More information can be found here https://www.amsterdam.nl/onderzoek/panels/stadspanel-amsterdam/resultaten/.

2 International migrants; proficiency in Dutch was sufficient to take part to the survey only available in Dutch.

3 Individuals with a migration background are defined as someone of whom at least one parent was born abroad.

4 For more information, see: www.projectmisty.org

5 During data collection, the Centraal Bureau for Statistiek (CBS) classified immigrants as “first generation” migrants, and children of at least one immigrant parent born in the Netherlands as “second generation” migrants. Non-migrants are counted as respondents with no migration background, indicating neither they nor their parents immigrated to the Netherlands.
